# Transcranial direct current stimulation combined with amantadine in repetitive mild traumatic brain injury in rats

**DOI:** 10.1186/s12868-022-00763-3

**Published:** 2022-12-11

**Authors:** Soo Jeong Han, Gahee Park, Jee Hyun Suh

**Affiliations:** Department of Rehabilitation Medicine, College of Medicine, Ewha Womans University, 1071 An-Yang-Cheon Ro, Yang-Cheon Gu, Seoul, Republic of Korea

**Keywords:** tDCS, Mild traumatic brain injury, Balance, Memory

## Abstract

**Background:**

Balance and memory deficits are common in patients with repetitive mild traumatic brain injury (mTBI).

**Objective:**

To investigate the combined effects of amantadine and transcranial direct current stimulation (tDCS) on balance and memory in repetitive mTBI rat models.

**Methods:**

In this prospective animal study, 40 repetitive mTBI rats were randomly assigned to four groups: tDCS, amantadine, combination of amantadine and anodal tDCS, and control. The tDCS group received four sessions of anodal tDCS for four consecutive days. The amantadine group received four intraperitoneal injections of amantadine for four consecutive days. The combination group received four intraperitoneal injections of amantadine and anodal tDCS for four consecutive days. Motor-evoked potential (MEP), rotarod test, and novel object test results were evaluated before mTBI, before treatment, and after treatment.

**Results:**

All groups showed significant improvements in the rotarod and novel object tests, particularly the combination group. The combination group showed a significant improvements in duration (p < 0.01) and maximal speed in the rotarod test (p < 0.01), as well as an improvement in novel object ratio (p = 0.05) and MEP amplitude (p = 0.05) after treatment. The combination group exhibited a significant increase in novel object ratio compared to the tDCS group (p = 0.04). The GFAP integral intensity of the left motor cortex and hippocampus was the lowest in the combination group.

**Conclusion:**

Combination treatment with amantadine and tDCS had positive effects on balance and memory recovery after repetitive mTBI in rats. Therefore, we expect that the combination of amantadine and tDCS may be a treatment option for patients with repetitive mTBIs.

## Introduction

In the United States, approximately 2.5 million cases of traumatic brain injury (TBI) occur each year, 75–90% of which are estimated to be mild [1, 2] Patients with mild TBI (mTBI) present not only cognitive symptoms such as memory impairment, reduced processing speed, and difficulty in focusing attention, but also motor symptoms such as difficulties in gait and balance [3, 4] A large proportion of patients with mTBI have suffered for an extended period because of the symptoms of mTBIs. In addition, when mTBI occurs repetitively, the prognosis is poor. Recent studies have demonstrated the cumulative effects of repetitive mTBI, which results in the excessive accumulation of tau protein [5] Previous studies have shown that repetitive mTBI can lead to an increased likelihood of developing neurodegenerative diseases such as Alzheimer’s disease, Parkinson’s disease (PD), or chronic traumatic encephalopathy [6] Accordingly, there is growing awareness of repetitive mTBI and a recognition of the need for effective treatment options for repetitive mTBI.

Transcranial direct current stimulation (tDCS) is a non-invasive treatment modality that modulates neural membrane potential by delivering subthreshold electrical currents to brain neuroinflammation [7] Anodal tDCS has been reported to increase neural plasticity, leading to motor improvements in a rat model of TBI [7]. Our previous study showed that anodal tDCS could significantly improve balance function in a repetitive mTBI rat model [8] However, only a few studies have investigated the effects of anodal tDCS on memory in repetitive mTBI cases.

Amantadine is a noncompetitive N-methyl-D-aspartate (NMDA) antagonist that may provide neuroprotection through the inhibition of excitatory glutamate receptors [9] Previous studies have shown that amantadine has the potential to improve motor and cognitive function in TBI rats [10] In contrast, another study reported no statistically significant effect of amantadine on cognitive function in subjects with TBI [11]

To date, there have been no studies on the combination therapy of amantadine and tDCS for the treatment of balance impairment and memory deficits in repetitive mTBI cases. Hence, this study aimed to investigate the effect of this combination therapy. We hypothesized that amantadine and tDCS have positive effects on balance and memory improvements in repetitive mTBI rats.

## Material and methods

This prospective, randomized animal study was approved by the Institutional Animal Care and Use Committee (approval number ESM13-0235). 4-week-old healthy male Sprague–Dawley rats (Orient Bio, Seongnam, Korea) weighing 80–90 g supplied by a single-source breeder were used in this study. 4-week-old rats were used in this study as this age corresponds to the teenagers and young adults in rats, which is a common age of mTBI occurrence in humans [12] Only male rats were used in this study as the hormonal levels could influence the cortical excitability and the neurotransmitter levels could affect the tDCS response [13–15] The male would receive more current at the cortex than the female due to the cortical bone structure [16] Furthermore, males make up a larger percentage of cases than females in mTBI [17] For these reasons, in this study, only male rats were included. The animals were under standard conditions with 12-h light–dark cycle, and had free access to tap water and regular rat chow. All animals received human care in compliance with the National Institutes of Health guidelines for the use of experimental animals. This study was carried out in compliance with the ARRIVE guidelines. This study was performed using protocols approved by the Institutional Animal Care and Use Committee (Approval number ESM13-0235).

### Repetitive mTBI models

mTBI was induced in rats using a modified weight-drop device and a protocol previously described by Tang et al [18] Through previous studies, we have demonstrated that the weight-drop device used in this study causes only mild traumatic brain injury without causing histological changes or imaging changes such as cerebral hemorrhage [8, 19, 20] The animals were anesthetized by intramuscular injection of tiletamine/zolazepam (10 mL/kg; zoletil^Ⓡ^, Vibac, France), and placed on the wooden platform of the device in the prone position. A 175 g steel weight was briefly dropped from a height of 30 cm through a polyvinyl chloride tube with an inner diameter of 11 mm terminating on the bregma of the rat. NCAA Concussion Study (1999–2001) showed the average interval between first and repeat concussion was 5.59 days. [21] This is a time interval equivalent to 0.89 h in rats. And atheletes reporting a history of 3 or more previous concussions were 3.0 times more likely to have an incident concussion than atheletes with no concussion history [22] Based on these previous studies, the mTBI procedure was performed 3 times at 1-h intervals for a repetitive mTBI model.

### Experimental design

**Fig. 1:**
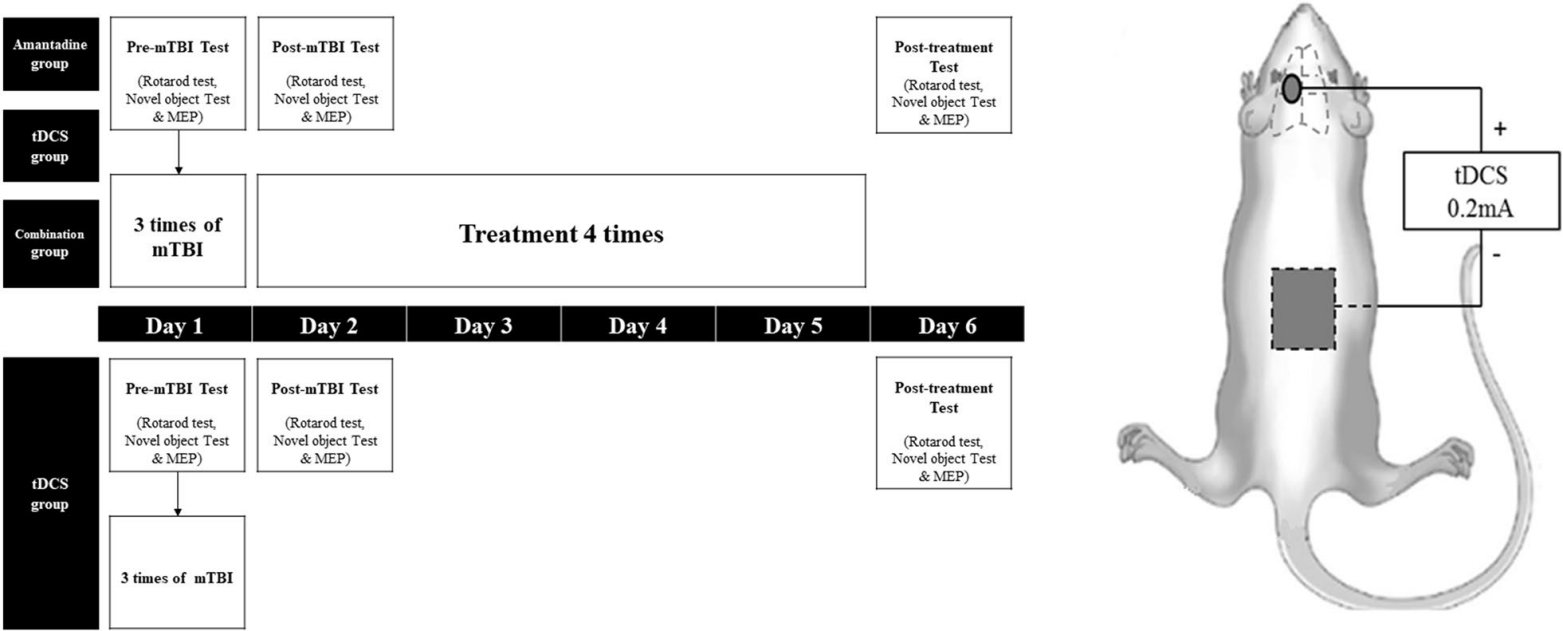
Experiment schedule and the position of electrodes during tDCS. **a** Experiment schedule. **b** The position of anodal and cathodal electrodes during anodal tDCS. A cup–shaped anodal electrode was placed on the scalp over the left motor cortex, and a rectangular rubber cathodal electrode was affixed to the abdomen

Forty rats underwent repetitive mTBIs (Day 1) and were randomly assigned to one of four groups: amantadine group (n = 10) that received intraperitoneal injections of amantadine alone, tDCS group (n = 10) that underwent anodal tDCS alone, combination of amantadine and anodal tDCS (amantadine + tDCS) group (n = 10) that underwent both amantadine intraperitoneal injections and anodal tDCS, and control group (n = 10) that did not undergo additional treatment. In the combination group, amantadine injection was administered first, followed by tDCS. All treatments were performed 4 times for four consecutive days (Days 2–5), once a day. The treatment and test schedules are shown in Fig. 1.

### Experimental procedure

The day after three inductions of mTBI (Day 2), amantadine injection and anodal tDCS were conducted (Fig. 1a). The amantadine and combination groups received daily intraperitoneal injections of amantadine hydrochloride (10 mg/kg; Sigma Chemical Co., St. Louis, MO, USA) [23]. Amantadine hydrochloride was dissolved in 0.9% sterile saline and administered. Amantadine hydrochloride was injected once a day for 4 days (Days 2–5). Ion combination group, amantadine injection was administered before tDCS application.

In tDCS and combination groups, anodal tDCS was performed under isoflurane-induced anesthesia (2% isoflurane in a 1:2 mixture of O_2_/N_2_O) [20] A tDCS was applied using a constant-current stimulator, PhoresorII^Ⓡ^ (IOMED, Salt Lake City, UT, USA). A constant direct current was delivered for 30 min at an intensity of 0.2 mA and a density of 0.255 mA/cm^2^ (0.2 mA/0.785 cm^2^). The fur around the bregma was removed to ensure tight attachment of the anodal electrode. A 1 cm-diameter cup–shaped anodal electrode was placed on the scalp over the left motor cortex, 3 mm to the left of the bregma, and 2 mm in front of the interaural line, using a high-conductivity fixation cream (Fig. 1b). A 30 mm × 30 mm rectangular rubber cathodal electrode was affixed to the abdomen [8, 20, 24]. Anodal tDCS was conducted once a day for 4 days (Day 2–5, Fig. 1a). tDCS was performed by a single experienced physiatrist.

### Measurements

#### Behavioral tests

In this study, two behavioral tests were performed; the rota rod test and the novel object test. These tests were conducted pre-mTBI (Day 1), post-mTBI (Day 2) and one day after the last session of treatment (Day 6), to eliminate anesthetic effects (Fig. 1a). For evaluation of the balance control and motor coordination, the rotarod test was used [25] A rat was placed on the rotarod treadmill. The rotation speed was started at 4 rpm and accelerated to 40 rpm over a 4 min period [7] The duration of the rolling rotarod before falling and the maximal speed on the rotarod were recorded and analyzed. Three trials were performed, and the average value was calculated. For the evaluation of the memory function, the modified version of the novel object test developed by Ennaceur and Delacour was used [10, 26, 27] Two identical objects were placed in an acryl room with black walls and floors, and the rats were allowed to explore for 5 min. One hour later, one of the objects was swapped for a different object, the rat was placed back in the room, and the time spent exploring each object was measured. The ratio between the times spent exploring the new object and the time spent exploring the familiar object was then compared. The objects were changed in every trial. In the first trial, the familiar object was a tetrahedron, and the new object was a cuboid. In the second trial, the familiar object was a cylinder, and the new object was a conical flask model. In the third trial, the familiar object was a narrow tetrahedron, and the new object was a narrow cuboid. A higher ratio meant that more time was spent exploring the new object, which was interpreted as a better memory function.

### Motor-evoked potentials (MEP)

To evaluate the functional integrity of the motor system, transcranial MEP was evaluated. MEPs are muscle action potentials elicited by transcranial magnetic brain stimulation [28] In this study, MEP measurements were evaluated pre-mTBI (day 1), post-mTBI (day 2), and post-tDCS (day 6) to evaluate the excitability of the corticospinal pathway (Fig. 1). MEP at the right tibialis anterior muscle of the hindlimb was evaluated. The MEP was recorded from the tibialis anterior muscle of the right hindlimb, which resulted from left motor cortex stimulation. The monopolar uninsulated stainless-steel active needle electrode was inserted into the belly of the tibialis anterior muscle, and the reference needle electrode was inserted into the distal part of the tibialis anterior muscle. The ground electrode was placed on an opposite footpad. The MEP test was performed using a Medtronic Keypoint^Ⓡ^ laboratory computer (Medtronic Inc., Jacksonville, FL, USA). The measurement settings were a sweep velocity of 5 ms with a sensitivity of 200 μV, and the bandpass filter setting was 20 Hz–10 kHz. Single-pulse transcranial magnetic stimulation was administered over the left motor cortex, which was anterior and left lateral to the bregma, with a figure-of-eight magnetic coil (diameter of one widening = 50 mm, peak magnetic field = 4.0Tesla) using a magnetic stimulator, Magstim^Ⓡ^ (Magstim Company, Whiteland, Wales, United Kingdom). The center of the coil was positioned on the left motor cortex, whose center was anterior and lateral to the bregma on the right side of the hindlimb, where the active needle electrode was inserted. A total of 20 MEPs were recorded at 10 s inter-stimulus intervals [29] TMS intensity was recorded as percent machine output (MO), with 100% corresponding to the maximal amplitude electrical current conducted through the magnetic coil. We set the stimulation intensity to 100% MO [20]. The intensity of the stimulation was maintained constant throughout the procedure. The average latency and largest peak-to-peak amplitude of the MEP waves were recorded and analyzed.

### Glial fibrillary acidic protein (GFAP) immunohistochemistry

GFAP is a protein found only in the central nervous system. GFAP levels are increased when astrocytes are damaged [30]. A low GFAP integral intensity indicates a decrease in reactive astrocytosis, which can play a neurotoxic role and aggravate neural death [31, 32] Previous study showed that GFAP could determine patients with mTBI with subtle injuries detected only through MRI [33] 30 days after all treatments and evaluations (Day 36), 12 rats (3 rats from each group were randomly selected) were euthanized by carbon dioxide inhalation using an approved standard protocol. The brain tissue washed PBS for removing the bound and unbound reagents/serum component. The fresh brain is fixed in 10% formalin to prevent deformation or deterioration due to autolysis. Formalin-fixed tissue undergoes tissue processing and then is embedded in paraffin wax to create the paraffin block. Embedding is important in preserving tissue morphology and giving the tissue support during sectioning. Brain slices were sectioned to a thickness of 4 μm. The tissues incubated using the free floating method. Immunohistochemical staining was performed to assess axonal damage and astrocytes. The slices were incubated with primary antibody against GFAP ( 1:500 dilution, Ab4674, Abcam, Cambridge, United Kingdom) at room temperature for 30 min, and with conjugated secondary antibodies (1: 200 dilution, Ab6877, Abcam, Cambridge, United Kingdom) for 20 min [20] After staining, the tissue sections were washed with running water and mounted using a universal mount (Dako, Carpinteria, CA, U.S.A.). Left motor cortex and hippocampus were assessed and averaged in a blinded manner. In this study, respectively one representative left motor cortex tissue slice and hippocampus slice were selected and analyzed in each brain. Total twelve left motor cortex slices and twelve hippocampal slices were included. After tissue sections were obtained. Immunohistochemical study was performed with Bond Max (Leica Biosystems, Newcastle, UK). The mean integral intensity of GFAP was calculated and analyzed. A computer-assisted image analysis program, AnalySIS^Ⓡ^ (Soft Imaging System, GmbH, Munster, Germany) was used to measure GFAP expression [20] Images were captured from left motor cortex and hippocampus. The software automatically changed the color of all immunolabeled elements beyond the threshold range into red pixels and changed the color of the rest of the image into gray pixels. The software then estimated the intensity of pure red pixels [20, 34]

### Statistical analysis

To verify the effects of mTBI, Wilcoxon signed-rank test was performed to compare the results of pre-mTBI and post-mTBI evaluation results for all rats. After treatment, to compare the effects among the four groups, each treatment effect was measured with changes in each parameter (post-treatment values minus post-mTBI (pre-treatment) values; △). The Kruskal–Wallis test was used to compare the effects between groups. If the Kruskal–Wallis test was positive, the Mann–Whitney test was used for comparison between the two groups. In addition, to analyze the within-group effectiveness of treatments, we used the Wilcoxon signed test. Statistical analysis was performed using SPSS version 21.0 (IBM SPSS, Armonk, NY, USA), and p-values of 0.05 or less, were considered statistically significant.

## Results

**Fig. 2:**
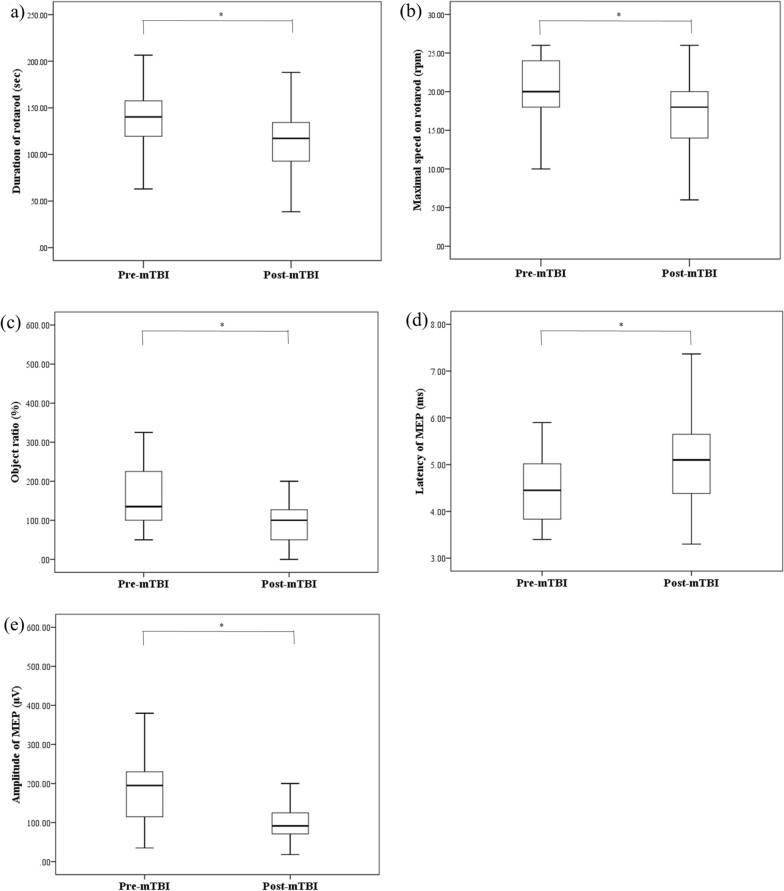
Box plot of the effects of repetitive mTBIs in behavioral tests and MEP. When comparing the pre-mTBI and postmTBI results in 40 repetitive mTBI rats, there were significant differences in all behavioral and MEP tests. (**a**) There were significant differences in the duration of rotarod between pre-mTBI and post-mTBI. (**b**) There were significant differences in the maximal speed on rotarod between pre-mTBI and post-mTBI. (**c**) There were significant differences in the object ratio between pre-mTBI and post-mTBI. (**d**) There were significant differences in the latency of MEP between pre-mTBI and post-mTBI. (**e**) There were significant differences in the amplitude of MEP between pre-mTBI and post-mTBI

When comparing the pre-mTBI and post-mTBI results in 40 repetitive mTBI rats, there were significant differences in all behavioral and MEP tests (Table 1, Fig. 2).

**Table 1:** The effects of repetitive mTBIs in behavioral tests and MEP. There were significant differences in all behavioral and MEP tests between pre-mTBI and post-mTBI

	Pre-mTBI	Post-mTBI	p-value
Duration on rotarod (sec)	136.61 ± 4.91	109.60 ± 6.40^a^	< 0.01
Maximal speed on rotarod (rpm)	20.60 ± 0.68	16.60 ± 5.75^a^	< 0.01
Object ratio (%)	182.30 ± 21.08	94.68 ± 8.77^a^	< 0.01
Latency of MEP (ms)	4.47 ± 0.11	5.04 ± 0.13^a^	< 0.01
Amplitude of MEP (μV)	194.93 ± 18.99	10.15 ± 8.20^a^	< 0.01

*mTBI* mild traumatic brain injury; results represent mean value ± standard error of mean

^a^ ≤ 0.05

### Behavioral tests

After treatment, the combination and tDCS groups showed significantly longer duration on rotarod (Combination, p < 0.01; tDCS, p < 0.01) and faster speed on rotarod test (Combination, p < 0.01; tDCS, p < 0.01) (Table 2). The Amantadine group showed no significant difference in the rotarod test between the post-mTBI and post-treatment values. The combination and amantadine groups also showed a significant increase in the object ratio of the novel object test (Combination, p ≤ 0.05; Amantadine, p ≤ 0.05) (Table 2). The tDCS group did not show a significant improvement in the novel object test after treatment. There was no significant improvement in the behavior of the control group.

**Table 2:** Behavioral and MEP test results of each groups

	Control			Amantadine			tDCS			Combination		
	Pre-mTBI	Post-mTBI	Post-treatment	Pre-mTBI	Post-mTBI	Post-treatment	Pre-mTBI	Post-mTBI	Post-treatment	Pre-mTBI	Post-mTBI	Post-treatment
Duration on rotarod (sec)	141.15 ± 10.06	118.95 ± 7.72	121.15 ± 9.88	133.50 ± 8.66	100.80 ± 11.41	131.55 ± 16.95	147.30 ± 4.88	102.75 ± 16.72	150.30 ± 16.10^**b**^	124.50 ± 13.65	115.90 ± 14.65	164.05 ± 18.64^**b**^
Maximal speed on rotarod (rpm)	21.00 ± 1.202	18.00 ± 0.99	17.80 ± 1.28	20.20 ± 1.245	15.40 ± 1.66	20.00 ± 2.13	22.40 ± 0.884	15.40 ± 2.39	22.20 ± 2.220^**b**^	18.80 ± 1.867	17.60 ± 2.06	24.20 ± 2.641^**b**^
Object ratio (%)	217.88 ± 60.17	94.98 ± 19.39	81.79 ± 11.92	170.40 ± 42.86	104.30 ± 21.66	156.89 ± 22.07	201.19 ± 39.02^**b**^	119.47 ± 11.81	149.11 ± 14.54	139.76 ± 17.98	59.98 ± 11.62	159.55 ± 14.82^**b**^
Latency of MEP (ms)	4.01 ± 0.17	5.20 ± 0.36	5.34 ± 0.25	4.29 ± 0.22	5.13 ± 0.18	5.50 ± 0.21	4.97 ± 0.22	4.97 ± 0.28	4.74 ± 0.23	4.59 ± 0.19	4.85 ± 0.26	4.56 ± 0.20
Amplitude of MEP (μV)	203.70 ± 33.75	92.40 ± 13.92	69.80 ± 13.27	133.70 ± 31.23	81.30 ± 10.52	94.60 ± 11.43	262.10 ± 52.32	131.40 ± 24.22	215.00 ± 30.23	180.20 ± 21.10	100.70 ± 11.00	123.60 ± 13.90^a^

The duration and maximal speed on rotarod were significantly improved in the combination and tDCS group. The object ratio of the novel object test was significant increased in the combination group and amantadine groups. Combination group showed significantly increased amplitude of MEP after treatments

^a^ ≤ 0.05 significant difference between post-mTBI and post-treatment by Wilcoxon signed rank test

^b^ ≤ 0.01 significant difference between post-mTBI and post-treatment by Wilcoxon signed rank test

**Fig. 3:**
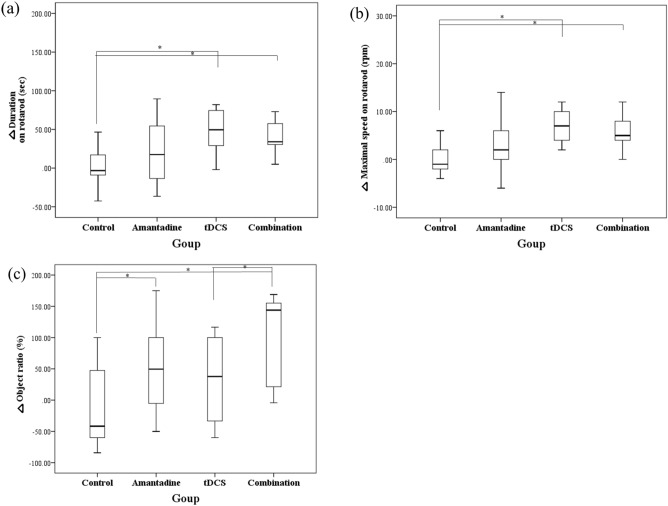
Comparison of the treatment effects on behavioral tests. **a** tDCS and combination groups showed significant increase of duration on rotarod compared with control group (p < 0.01). **b** tDCS and combination groups showed significant increase of maximal speed on rotarod compared with control group (p < 0.01). **c** In novel object recognition test, combination group showed a higher increase of object ratio than control and tDCS group (p < 0.01, p = 0.04). Amantadine group showed a significant increase of object ratio compared with control group (p = 0.04) tDCS; transcranial direct current stimulation, *p ≤ 0.05

There were significant differences among the four groups in the rotarod test (△duration, p = 0.01; △maximal speed, p < 0.01) and novel object test (p = 0.01) (Table 3, Fig. 3). In rotarod test, the combination and tDCS groups showed significantly longer △duration on rotarod (combination, p < 0.01; tDCS, p < 0.01) (Fig. 3a) and significantly faster △maximal speed on rotarod (combination, p < 0.01; tDCS, p < 0.01) (Fig. 3b) than the control group. The amantadine group showed no significant difference compared with the control group in the rotarod test. There was no significant difference between the combination group and the other treatment groups in the rotarod test. In novel object test, combination group showed significantly greater increase of △object ratio than the control (p < 0.01) and tDCS groups (p = 0.04) (Fig. 3c). The amantadine group also showed a significantly greater increase in △object ratio than the control group (p = 0.04) (Fig. 3c). The combination group did not show a significant difference compared to the amantadine group. The tDCS group showed no significant difference compared to the control and amantadine groups in the novel object test.

**Table 3:** Calculated differences (post-treatment values minus post-mTBI (pre-treatment) values; △) between pre-treatment (post-mTBI) and post-treatment evaluations

	Control	Amantadine	tDCS	Combination
**△Duration on rotarod**^**b**^(sec)	2.20 ± 8.43^**d**^	30.75 ± 20.34	47.55 ± 8.89^**a**^	48.15 ± 12.25^**a**^
**△Maximal speed on rotarod**^**b**^(rpm)	− 0.20 ± 1.05^**d**^	4.60 ± 2.75	6.80 ± 1.08^**a**^	6.60 ± 1.81^**a**^
**△Object ratio**^**b**^ (%)	− 13.18 ± 20.28^**c**^	52.58 ± 21.29^**a**^	29.64 ± 22.28	99.58 ± 23.13^**a**^**,**^**d**^
**△**MEP latency(ms)	0.15 ± 0.41	0.37 ± 0.27	**− **0.24 ± 0.26	**− **0.29 ± 0.23
**△MEP amplitude**^**b**^(μV)	− 22.60 ± 17.72^**d**^	13.30 ± 15.69^**d**^	83.60 ± 41.30^**a**^**,**^**c**^	22.90 ± 9.54^**d**^

In rotarod test, the combination and tDCS groups showed significantly longer △duration on rotarod and significantly faster △maximal speed on rotarod than the control group. In novel object test, combination group showed significantly greater increase of △object ratio than the control and tDCS groups. In MEP study, the tDCS group showed a significantly greater increase of △MEP amplitude compared to the other groups. *mTBI* mild traumatic brain injury, *tDCS* transcranial direct current stimulation, *MEP* motor-evoked potential. Results represent mean value ± standard error of mean

^a^ ≤ 0.05 significant difference compared with the control group by MannWhitney test

^b^ ≤ 0.05 significant difference among the four groups by Kruskal-Wallis test

^C^ ≤ 0.05 significant difference compared with the amantadine group by MannWhitney test

^d^ ≤ 0.05 significant difference compared with the tDCS group by Mann–Whitney test

### MEP test

Combination group showed significantly increased amplitude of MEP after treatments (p = 0.05) (Table 2). In the tDCS group, the amplitude of MEP showed an increasing tendency after treatment compared to before treatment (p = 0.06) (Table 2). Amantadine and control groups showed no significant changes in MEP amplitude after treatment. There were no significant changes in the MEP latency in any of the groups.

**Fig. 4:**
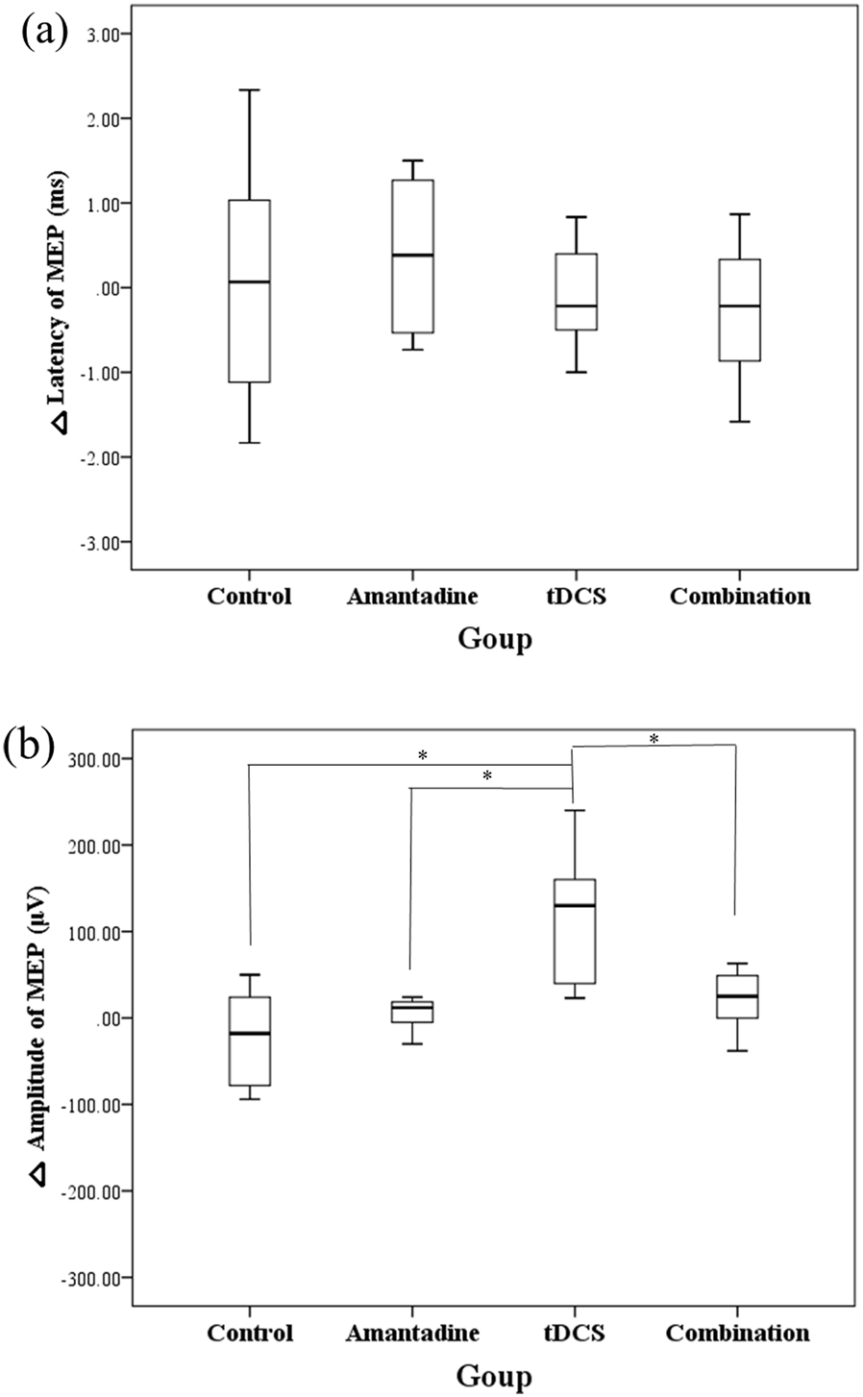
Comparison of the treatment effects in MEP test. **a** There was no significant difference in the aspect of MEP latency. **b** MEP amplitude was significantly increased in tDCS group, compared with control, amantadine and combination groups (p ≤ 0.05) MEP; motor-evoked potential, tDCS; transcranial direct current stimulation, *p ≤ 0.05

There was a significant difference in △MEP amplitude among the four groups (p = 0.02) (Table 3, Fig. 4(a)). In each group comparisons, the tDCS group showed a significantly greater increase of △MEP amplitude compared to the other groups (control, p = 0.01; amantadine, p = 0.02; combination, p = 0.04) (Fig.4b). The combination group showed a tendency of a greater increase of △MEP amplitude compared with the control group (p = 0.09) (Fig. 4b). The amantadine group showed no significant difference compared with the control group. There was no significant difference in △MEP latency between the groups (Fig. 4a).

### Immunohistochemical findings

**Fig. 5:**
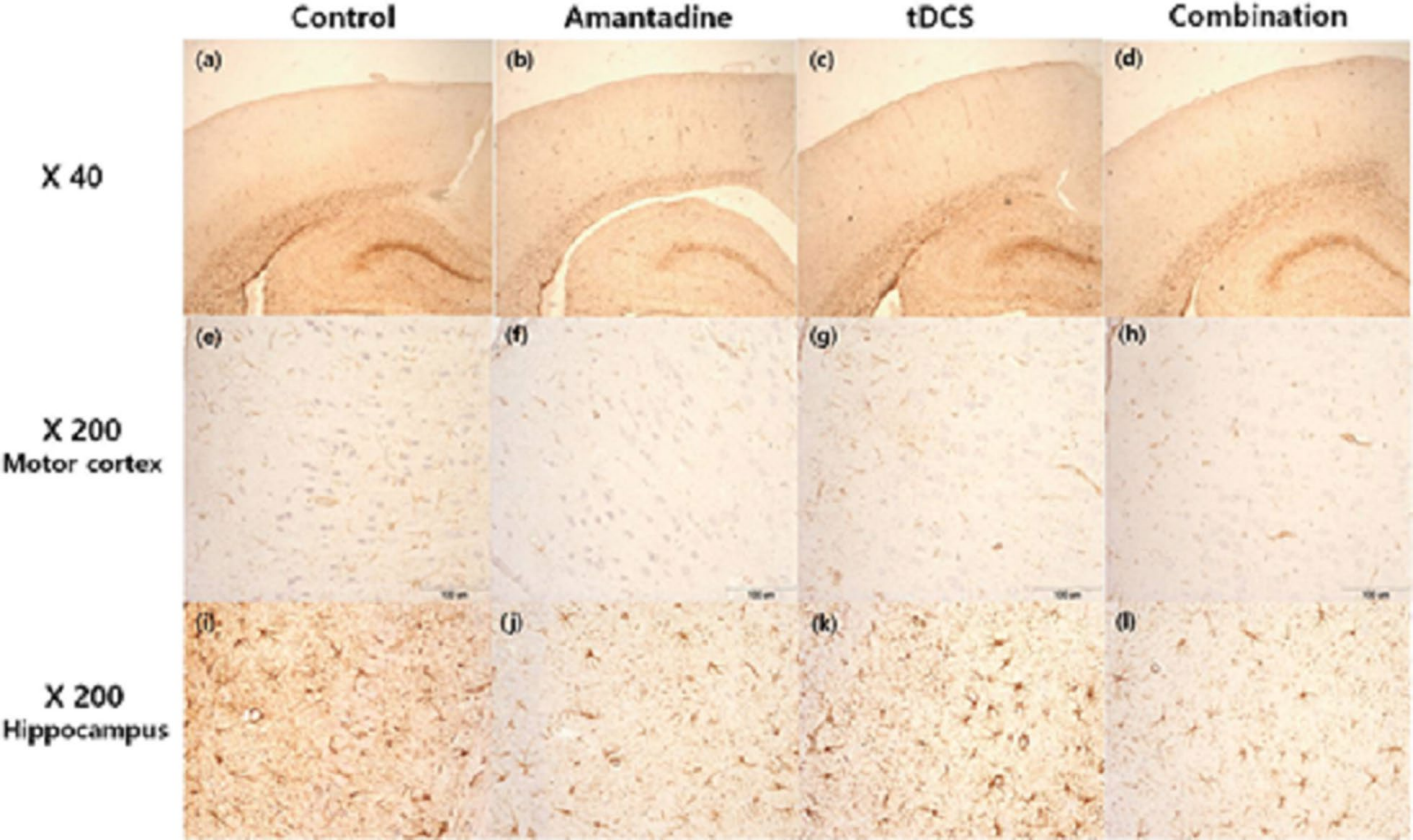
Representative photographs of the left motor cortex and hippocampus with GFAP immunostain labeling. (**a**–**d**): ×40 magnification, (**e**)–(**l**): ×200 magnification, (**e**)–(**h**): motor cortex, (**i**)–(**l**): hippocampus, (**a**, **e**, **i**): control group, (**b**, **f**, **j**): amantadine group, (**c**, **g**, **k**): tDCS group, (**d**, **h**, **l**): combination group). The calibration bar is 100 μm

**Fig. 6:**
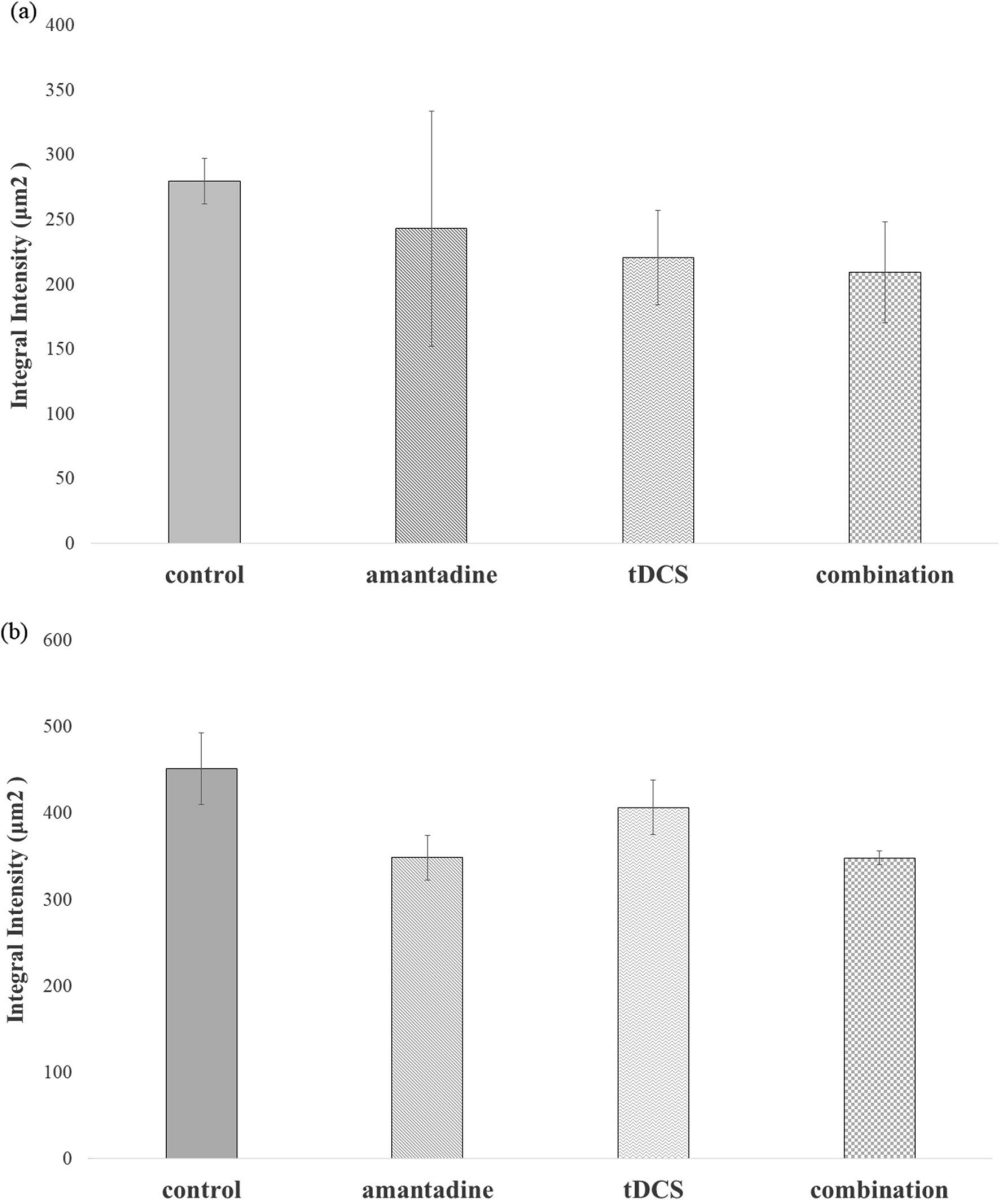
The integral intensity of GFAP in left motor cortex and hippocampus. **a** In the left motor cortex, the integral intensity was the lowest in the combination group, followed by the tDCS, amantadine, and control groups **b** In the left hippocampus, the integral intensity of GFAP was the lowest in the combination group, followed by the amantadine, tDCS, and control groups

Three brains in each four groups, a total of 12 brains were underwent the immunohistochemical study with GFAP stain. Reactive astrocytosis was observed in all slides, and the integral intensities were measured. The integrated intensities of GFAP in the treatment groups were lower than those in the control groups. In the left motor cortex, the integral intensity was the lowest in the combination group, followed by the tDCS, amantadine, and control groups (Fig. 5, 6a). The integral intensity of GFAP at the motor cortex was 209.43 ± 39.18 μm^2^ in the combination group, 220.74 ± 36.39 μm^2^ in tDCS group, 243.00 ± 90.73 μm^2^ in amantadine group, and 279.84 ± 17.59 μm^2^ in control group (Fig. 5, 6a). In the left hippocampus, the integral intensity of GFAP was the lowest in the combination group, followed by the amantadine, tDCS, and control groups (Fig. 5, 6b).

## Discussion

In this study, the amantadine group showed an improvement in object ratio. The tDCS group showed improvements in duration and maximal speed in the rotarod test, as well as amplitude in the MEP study. The combination group, which received both amantadine and tDCS treatments, showed significant improvements in duration and maximal speed in the rotarod test, as well as object ratio, when compared to the control group. In addition, the combination group showed the greatest improvements in the GFAP integral intensities of the left motor cortex and hippocampus compared to the tDCS and amantadine groups, respectively. This study suggests that tDCS and combination treatment may be beneficial for improving motor coordination and balance function, while amantadine and combination treatment may help improve memory function. Hence, the combination treatment of tDCS and amantadine has positive effects not only on balance, but also on memory function in repetitive mTBI rat models.

According to the pathophysiology of TBI, NMDA receptors are activated following TBI, which results in the acceleration of calcium accumulation, leading to neuronal death [35]. In addition, neurotransmitters are also altered, especially in the glutamatergic system, which is also related to NMDA receptors. Alterations in neurotransmitter levels can cause neuronal death and balance dysfunction [36]. Therefore, amantadine, an NMDA receptor antagonist, may be effective in reducing neural damage. Amantadine, a nonselective NMDA receptor antagonist, is a dopaminergic agent that presynaptically facilitates the release of dopamine and inhibits its reuptake, thereby increasing the concentration of dopamine in the synaptic cleft. Amantadine also has a direct postsynaptic effect on dopamine receptors, increasing their density and/or altering their configuration. Previous studies have shown that amantadine improves cognitive function related to arousal, memory, and aggression in TBI [37] Based on this mechanism, the object ratio of the novel object test in the amantadine and the combination groups in this study may improve.

mTBI is responsible for a 56% higher risk of developing PD [38] Amantadine is currently used in the management of gait disorders and balance disturbance mechanisms in patients with PD through dopaminergic and non-dopaminergic mechanisms [39] Amantadine has been shown to stabilize the NMDA receptor channel in the closed conformation, thus antagonizing this receptor [40] Amantadine also increases extracellular dopamine levels, which may be due to NMDA receptor inhibition [41] Additionally, studies on rat pheochromocytoma suggest that amantadine may stimulate gene expression of L-amino acid decarboxylase, the enzyme responsible for converting levodopa to dopamine [42]. The improved balance of the combination group compared to the tDCS group in this study may be attributed to this mechanism of amantadine.

Anodal tDCS at the motor cortex (M1) improved the duration and maximal speed in the rotarod test, as well as amplitude in the MEP study. This is consistent with the results of our previous study [8]. In our previous study, anodal tDCS over M1 improved balance function by activating the corticospinal and pyramidal tract neurons in repetitive mTBI rat models [8].

In this study, the amantadine and combination groups showed a significant improvement in object ratio in the novel object test compared to the control group. Although not statistically significant, the greatest improvement was observed in the combination group. This suggests that anodal tDCS may have a positive effect on memory. A previous study showed that anodal tDCS over the M1 area enhanced visual recognition memory [43]. One possible mechanism of memory improvement by anodal tDCS over M1 is that such effects might be attributed to the remote effects of stimulating the primary motor cortex using tDCS, which resulted in influencing prefrontal regions [44] that play significant roles in memory, such as the premotor cortex, supplementary motor area,[45, 46] dorsolateral prefrontal cortex,[47] and cerebellum [48]. In this study, tDCS had a positive effect on memory improvement in a repetitive mTBI rat model through this mechanism.

In this study, the integral intensity of GFAP at the motor cortex and hippocampus were lower in the treatment group. A previous study demonstrated that amantadine prevents neuronal death induced by various toxins. Amantadine protects the retinal ganglion, nucleus basalis magnocellularis, and cortical and mesencephalic neurons from NMDA-induced toxicity [49–52]. Further, anodal tDCS can provoke neuroplasticity in repetitive mTBI rat models [20]. In a previous study, tDCS has been shown to reduce neuronal death resulting from transient cerebral ischemia. Thus, amantadine and tDCS had a preventive and positive effect on neuronal death, suggesting that the integral intensity was the lowest in the combination group.

## Limitations

The present study has several limitations. First, we did not conduct follow-up evaluations. The long-term effects are important for the application of treatments in clinical settings. Further studies involving long-term treatment and follow-up are required. Second, only a small number of repetitive mTBI rat models were included.

## Conclusion

This study demonstrated that combination treatment with amantadine and tDCS has positive effects on balance and memory recovery after repetitive mTBI in rats. In particular, the combination treatment group may have beneficial effects on balance and memory function compared to the control group. Amantadine acts as an NMDA receptor antagonist that reduces neural damage after mTBI. tDCS contributes to balance recovery by increasing motor cortex excitability. Therefore, we expect that the combination of amantadine and tDCS may be a treatment option for patients with repetitive mTBIs.

## Data Availability

Data will be available upon reasonable request to the corresponding author.

## Abbreviations

mTBI: Mild traumatic brain injury
tDCS: Transcranial direct current stimulation
NMDA: N-methyl-D-aspartate
MEP: Motor-evoked potential
GFAP: Glial fibrillary acidic protein
M1: Motor cortex

## References

[1] Prince C, Bruhns ME (2017). Evaluation and treatment of mild traumatic brain injury: the role of neuropsychology. Brain Sci.

[2] Taylor CA, Bell JM, Breiding MJ, Xu L (2017). Traumatic brain injury-related emergency department visits, hospitalizations, and deaths—United States, 2007 and 2013. MMWR Surveill Summ.

[3] Parker TM, Osternig LR, van Donkelaar P, Chou LS (2007). Recovery of cognitive and dynamic motor function following concussion. Br J Sports Med.

[4] Kushner D (1998). Mild traumatic brain injury: toward understanding manifestations and treatment. Arch Intern Med.

[5] Edwards G, Zhao J, Dash PK, Soto C, Moreno-Gonzalez I (2020). Traumatic brain injury induces tau aggregation and spreading. J Neurotrauma.

[6] VanItallie TB (2019). Traumatic brain injury (TBI) in collision sports: possible mechanisms of transformation into chronic traumatic encephalopathy (CTE). Metabolism.

[7] Yoon KJ, Lee YT, Chae SW, Park CR, Kim DY (2016). Effects of anodal transcranial direct current stimulation (tDCS) on behavioral and spatial memory during the early stage of traumatic brain injury in the rats. J Neurol Sci.

[8] Park G, Suh JH, Han SJ (2021). Transcranial direct current stimulation for balance and gait in repetitive mild traumatic brain injury in rats. BMC Neurosci.

[9] Zafonte RD, Lexell J, Cullen N (2000). Possible applications for dopaminergic agents following traumatic brain injury: part 1. J Head Trauma Rehabil.

[10] Huang EY, Tsui PF, Kuo TT (2014). Amantadine ameliorates dopamine-releasing deficits and behavioral deficits in rats after fluid percussion injury. PLoS ONE.

[11] Schneider WN, Drew-Cates J, Wong TM, Dombovy ML (1999). Cognitive and behavioural efficacy of amantadine in acute traumatic brain injury: an initial double-blind placebo-controlled study. Brain Inj.

[12] Holm L, Cassidy JD, Carroll LJ, Borg J (2005). Neurotrauma task force on mild traumatic brain injury of the WHOCC summary of the WHO collaborating centre for neurotrauma task force on mild traumatic brain injury. J Rehabil Med..

[13] Smith MJ, Keel JC, Greenberg BD (1999). Menstrual cycle effects on cortical excitability. Neurology.

[14] Epperson CN, Haga K, Mason GF (2002). Cortical gamma-aminobutyric acid levels across the menstrual cycle in healthy women and those with premenstrual dysphoric disorder: a proton magnetic resonance spectroscopy study. Arch Gen Psychiatry.

[15] Batra NA, Seres-Mailo J, Hanstock C (2008). Proton magnetic resonance spectroscopy measurement of brain glutamate levels in premenstrual dysphoric disorder. Biol Psychiatry.

[16] Russell M, Goodman T, Wang Q, Groshong B, Lyeth BG (2014). Gender differences in current received during transcranial electrical stimulation. Front Psychiatry.

[17] Laker SR (2011). Epidemiology of concussion and mild traumatic brain injury. PM R.

[18] Tang YP, Noda Y, Hasegawa T, Nabeshima T (1997). A concussive-like brain injury model in mice (I): impairment in learning and memory. J Neurotrauma.

[19] Kim HJ, Han SJ (2017). A simple rat model of mild traumatic brain injury: a device to reproduce anatomical and neurological changes of mild traumatic brain injury. PeerJ.

[20] Kim HJ, Han SJ (2017). Anodal transcranial direct current stimulation provokes neuroplasticity in repetitive mild traumatic brain injury in rats. Neural Plast.

[21] McCrea M, Broglio S, McAllister T (2020). Return to play and risk of repeat concussion in collegiate football players: comparative analysis from the NCAA concussion study (1999–2001) and CARE consortium (2014–2017). Br J Sports Med.

[22] Guskiewicz KM, McCrea M, Marshall SW (2003). Cumulative effects associated with recurrent concussion in collegiate football players: the NCAA concussion study. JAMA.

[23] Dixon CE, Kraus MF, Kline AE (1999). Amantadine improves water maze performance without affecting motor behavior following traumatic brain injury in rats. Restor Neurol Neurosci.

[24] Kim SJ, Kim BK, Ko YJ, Bang MS, Kim MH, Han TR (2010). Functional and histologic changes after repeated transcranial direct current stimulation in rat stroke model. J Korean Med Sci.

[25] Schaar KL, Brenneman MM, Savitz SI (2010). Functional assessments in the rodent stroke model. Exp Transl Stroke Med.

[26] Grayson B, Leger M, Piercy C, Adamson L, Harte M, Neill JC (2015). Assessment of disease-related cognitive impairments using the novel object recognition (NOR) task in rodents. Behav Brain Res.

[27] Ennaceur A, Delacour J (1988). A new one-trial test for neurobiological studies of memory in rats 1: behavioral data. Behav Brain Res.

[28] Grunhaus L, Polak D, Amiaz R, Dannon PN (2003). Motor-evoked potential amplitudes elicited by transcranial magnetic stimulation do not differentiate between patients and normal controls. Int J Neuropsychopharmacol.

[29] Luft AR, Kaelin-Lang A, Hauser TK, Cohen LG, Thakor NV, Hanley DF (2001). Transcranial magnetic stimulation in the rat. Exp Brain Res.

[30] Lee HHLW, Seo HG, Han D, Kim Y, Oh BM (2017). Current state and prospects of development of blood-based biomarkers for mild traumatic brain injury. Brain Neurorehabil.

[31] Verkhratsky A, Olabarria M, Noristani HN, Yeh CY, Rodriguez JJ (2010). Astrocytes in Alzheimer’s disease. Neurotherapeutics.

[32] Eng LF, Ghirnikar RS (1994). GFAP and astrogliosis. Brain Pathol.

[33] Gill J, Latour L, Diaz-Arrastia R (2018). Glial fibrillary acidic protein elevations relate to neuroimaging abnormalities after mild TBI. Neurology.

[34] Limoa E, Hashioka S, Miyaoka T (2016). Electroconvulsive shock attenuated microgliosis and astrogliosis in the hippocampus and ameliorated schizophrenia-like behavior of Gunn rat. J Neuroinflammation.

[35] Giza CC, Hovda DA (2001). The neurometabolic cascade of concussion. J Athl Train.

[36] Sanders MJ, Sick TJ, Perez-Pinzon MA, Dietrich WD, Green EJ (2000). Chronic failure in the maintenance of long-term potentiation following fluid percussion injury in the rat. Brain Res.

[37] Stelmaschuk S, Will MC, Meyers T (2015). Amantadine to treat cognitive dysfunction in moderate to severe traumatic brain injury. J Trauma Nurs..

[38] Gardner RC, Byers AL, Barnes DE, Li Y, Boscardin J, Yaffe K (2018). Mild TBI and risk of Parkinson disease: a chronic effects of neurotrauma consortium study. Neurology.

[39] Parkes JD, Calver DM, Zilkha KJ, Knill-Jones RP (1970). Controlled trial of amantadine hydrochloride in Parkinson’s disease. Lancet.

[40] Blanpied TA, Clarke RJ, Johnson JW (2005). Amantadine inhibits NMDA receptors by accelerating channel closure during channel block. J Neurosci.

[41] Mizoguchi K, Yokoo H, Yoshida M, Tanaka T, Tanaka M (1994). Amantadine increases the extracellular dopamine levels in the striatum by re-uptake inhibition and by N-methyl-D-aspartate antagonism. Brain Res.

[42] Bennett VL, Juorio AV, Li XM (1999). Possible new mechanism for the antiparkinsonian effect of amantadine. J Psychiatry Neurosci.

[43] Bashir S, Bamugaddam A, Alasheikh M (2022). Anodal transcranial direct current stimulation (tDCS) over the primary motor cortex (M1) enhances motor response inhibition and visual recognition memory. Med Sci Monit Basic Res.

[44] Lefaucheur JP, Andre-Obadia N, Antal A (2014). Evidence-based guidelines on the therapeutic use of repetitive transcranial magnetic stimulation (rTMS). Clin Neurophysiol.

[45] Chein JM, Fiez JA (2001). Dissociation of verbal working memory system components using a delayed serial recall task. Cereb Cortex.

[46] Paulesu E, Frith CD, Frackowiak RS (1993). The neural correlates of the verbal component of working memory. Nature.

[47] Bashir S, Al-Hussain F, Hamza A, Asim Niaz T, Albaradie R, Habib SS (2019). Cognitive function assessment during 2 mA transcranial direct current stimulation in DLPFC in healthy volunteers. Physiol Rep.

[48] Marvel CL, Desmond JE (2010). Functional topography of the cerebellum in verbal working memory. Neuropsychol Rev.

[49] Wenk GL, Danysz W, Mobley SL (1995). MK-801, memantine and amantadine show neuroprotective activity in the nucleus basalis magnocellularis. Eur J Pharmacol.

[50] Chen HS, Pellegrini JW, Aggarwal SK (1992). Open-channel block of N-methyl-D-aspartate (NMDA) responses by memantine: therapeutic advantage against NMDA receptor-mediated neurotoxicity. J Neurosci.

[51] Lustig HS, Ahern KV, Greenberg DA (1992). Antiparkinsonian drugs and in vitro excitotoxicity. Brain Res.

[52] Weller M, Finiels-Marlier F, Paul SM (1993). NMDA receptor-mediated glutamate toxicity of cultured cerebellar, cortical and mesencephalic neurons: neuroprotective properties of amantadine and memantine. Brain Res.

